# Association of *Omentin* rs2274907 and *FTO* rs9939609 gene polymorphisms with insulin resistance in Iranian individuals with newly diagnosed type 2 diabetes

**DOI:** 10.1186/s12944-019-1085-5

**Published:** 2019-06-14

**Authors:** Amirhosein Khoshi, Mehdi Kaffash Bajestani, Habibesadat Shakeri, Golnaz Goodarzi, Fatemeh Azizi

**Affiliations:** 1 Department of Clinical Biochemistry, School of Medicine, North Khorasan University of Medical Sciences, Arkan roadway, Bojnurd, IR Iran; 2 Educational Development Center, North Khorasan University of Medical Sciences, Bojnurd, IR Iran; 3 Clinical Endocrinology, Department of Endocrinology and Internal Medicine, Imam Hassan Hospital, North Khorasan University of Medical Sciences, Bojnurd, Iran; 4 Student Research Committee, North Khorasan University of Medical Sciences, Bojnurd, IR Iran

**Keywords:** Type 2 diabetes, Insulin resistance, Omentin, Fat mass-and obesity associated (FTO), Gene polymorphism

## Abstract

**Background:**

Insulin resistance (IR) and fat accumulation in visceral adipose tissue are key players in developing type 2 diabetes (T2D). Several adipose tissue derived-gene polymorphisms are related to higher body mass index (BMI), insulin resistance and T2D. The association of *omentin* rs2274907 (Val109Asp) and *fat-mass and obesity-associated (FTO)* rs9939609 gene polymorphisms with overweight/obesity and T2D is controversial. The aim of this study was to determine the association between *omentin* Val109Asp and *FTO* rs9939609 polymorphisms and insulin resistance in newly-diagnosed T2D patients.

**Methods:**

The case-control study included 83 newly-diagnosed T2D patients and 85 healthy matched controls, aged 20–80 years. Fasting blood glucose and insulin levels were measured by the enzymatic method and enzyme-linked-immunosorbent assay, respectively. Insulin resistance was calculated using the homeostasis model assessment (HOMA) index. Genotyping was examined using the polymerase chain reaction-restriction fragment length polymorphism (PCR-RFLP).

**Results:**

There are significant differences between both *omentin* Val109Asp and *FTO* rs9939609 polymorphisms and studied individuals (*P* = 0.011 and *P* = 0.0001, respectively). Both genetic polymorphisms of *omentin* Val109Asp and *FTO* rs9939609 (T/A) are significantly related to higher HOMA index (*P* = 0.030 and *P* = 0.046, respectively). However, *omentin* Val109Asp polymorphism was only related to individuals who were overweight/obese. Additionally, both *omentin* Val109Asp and *FTO* rs9939609 polymorphisms were significantly positively correlated to familial history of diabetes (*P* = 0.046 and *P* = 0.024, respectively).

**Conclusions:**

*Omentin* V109D and *FTO* rs9939609 genetic variations may change insulin metabolism and have key roles in developing T2D through insulin resistance. Thus, the evaluation of these polymorphic regions may be helpful for predicting type 2 diabetes.

## Background

Type 2 diabetes mellitus (T2D) as the most common metabolic disorder arises from the impaired function of pancreatic beta cells and/or decreased sensitivity of peripheral tissues to insulin leading to insulin resistance (IR) and hyperglycemia. These defects might be associated with genetic and environmental factors that cause impairments in glucose, lipids, and amino acid metabolism [1, 2]. The IR is an important feature in T2D and is also implicated in other conditions, such as obesity and hypertension [3].

Obesity and overweight are defined as the body mass index (BMI) of ≥30 and ≥ 25 kg/m^2^, respectively. These conditions result from adipose tissue accumulation, especially in visceral (omental) and subcutaneous fats, and can change the level of adipokines. Obesity and overweight are closely related to diverse metabolic disorders, including IR, T2D, hypertension, dyslipidemia, and atherosclerosis [4, 5]. Based on the evidence, both lifestyle issues and genetic factors are involved in predisposing individuals to obesity [6, 7].

Omentin (also known as intelectin: *ITLN*) is one of the most major visceral fat adipokines expressed by genes 1 and 2. [8]. This adipokine is predominantly expressed in the vascular cells of the adipose tissue stroma, in addition to the epicardial fat, lungs, ovary, and placenta [4]. Expression of both *Omentin 1* and *Omentin 2* genes in visceral adipose tissue decreased with obesity [5]. The Omentin 1 is the main form of Omentin in circulation, and its exact biological role is still not well known [4, 9]. A number of studies have shown that Omentin enhances glucose uptake in human adipocytes by increasing insulin sensitivity [8, 10]. In addition, some studies indicated that plasma Omentin 1 level is inversely correlated with BMI, waist circumference, and IR as measured by homeostasis model assessment (HOMA). Moreover, there are data showing a positive correlation between Omentin and the levels of adiponectin and high-density lipoprotein (HDL) [5, 8].

It has been demonstrated in the literature that A326T (rs2274907) single nucleotide miss-sense polymorphism in the exon-4 of the *Omentin 1* gene, which substitutes valine instead of aspartate at position 109 (Val109Asp or V109D), could be accompanied by T2D and obesity [4, 11, 12]. Structurally, position 109 is located outside the fibrinogen domain of Omentin protein. Therefore, Val109Asp and other sequence variations can lead to a real disease-causing mutation [11].

On the other hand, based on a body of evidence, the common rs9939609 polymorphism in the *fat mass- and obesity-associated (FTO*) gene is associated with higher BMI, risk of obesity, and subsequent T2D in different populations [12–18].

Sequence analysis of FTO protein has predicted a 2-oxoglutarate oxygenase activity with a central role for transcriptional regulation during the hypoxic response [19, 20]. Moreover, histone demethylation has been found [21], particularly in hypothalamic nuclei, which control energy balance and arcuate nucleus that is regulated by the feeding-fasting process [22].

The polymorphisms of *Omentin* Val109Asp (rs2274907) and *FTO* rs9939609 genes are of high importance in overweight, obesity, and T2D. Moreover, T2D is positively associated with visceral adipose tissue hypertrophy. With this background in mind, the present study was conducted to investigate the simultaneous presence of *Omentin* Val109Asp and *FTO* rs9939609 polymorphisms in the Iranian population with newly diagnosed T2D and their relationships with IR.

## Materials and methods

### Study population

A case-control study was performed in Bojnurd, Northeast of Iran, during December 2016–June 2018. Informed consents were obtained from the individuals included in the study. The study protocol conforms to the ethical guidelines of the Declaration of Helsinki as reflected in a prior approval granted by the Human Research Ethics Committee of North Khorasan University of Medical Sciences in Bojnurd (ethics code of IR.nkums.REC.1396.38).

The eligible participants corresponded to a total of 168 individuals referring to the clinical laboratory of Imam Reza Hospital of Bojnurd city, including 83 cases with T2D and 85 healthy people without any complications considered as the control group. The two groups were matched in terms of age and gender. The study participants had an age range of 20–80 years with no history of hyperglycemia. Based on the clinical and laboratory investigations performed by an endocrinologist, 83 patients with T2D were selected as cases [23]. The case group had a fasting blood sugar level (FBS) of > 7 mmol/L as examined in two different times. In addition, 85 individuals with a normal level of FBS were considered as the control group.

The structured questionnaire covered such information as age, gender, smoking habits, BMI, familial history of diabetes, and history of medication usage, especially antidiabetic, lipid-lowering, and antihypertensive agents. The exclusion criteria included a history of hyperglycemia, or hepatic, cardiovascular, rheumatologic, renal, and thyroid diseases, as well as taking antihypertensive, anti-diabetic, lipid-lowering, and anti-inflammatory medications 1 month prior to sampling.

### Laboratory and molecular diagnosis

The FBS, triglyceride (TG), total cholesterol (TC), HDL cholesterol (HDL-C), and low-density lipoprotein cholesterol (LDL-C) were measured by enzymatic methods (Pars Azmun and Pishtaz Teb, Iran) using a biochemistry autoanalyzer (Dirui, China). Fasting serum insulin levels were also detected by enzyme-linked immunosorbent assay (ELISA) method (Demeditec Diagnostics GmbH, Germany) using a plate reader (BioTek, USA).

The HOMA-IR developed by Matthews et al. [24] is the most frequently employed technique both in clinical practice and epidemiological studies. Therefore, in the current study, IR assessment was carried out based on this model [25]. In this regard, HOMA-IR was calculated using the following equation: HOMA-IR = (Fasting insulin × Fasting glucose)/22.5 [26].

To analyze gene polymorphisms, blood samples were collected in ethylenediaminetetraacetic acid (EDTA)-coated vacuum tubes and stored at − 20 °C. The genomic deoxyribonucleic acid (DNA) was extracted using Proteinase K and column method according to the protocol of the manufacturer (Genet Bio, Korea). Afterwards, the DNA samples were randomly assessed by ultraviolet (UV) bio-spectrophotometer (Lambda, Japan), and their purity was checked by A260/ A280 ratio.

*Omentin* Val109Asp (V109D) and *FTO* rs9939609 gene polymorphisms were determined by polymerase chain reaction (PCR) using PreMix tubes (Genet Bio, Korea), followed by restriction fragment length polymorphism analysis (RFLP).

The PCR was used to amplify the amplicons of 471 and 182 bp for single-nucleotide polymorphisms (SNPs) in the exon-4 of *Omentin* and intron-1 of *FTO*, respectively. The oligonucleotide primers, namely F: 5′-GAGCCTTTAGGCCATGTCTCT-3′ and R: 5′-CTCTCCTTCTTCTCCAGCCCAT-3′, were utilized for *Omentin* Val 109 Asp polymorphism. In addition, we applied the primers F: 5′-AACTGGCTCTTGAATGAAATAGGATTCAGA-3′ and R: 5′- AGAGTAACAGAGACTATCCAAGTGCAGTAC-3′ for *FTO* rs9939609 polymorphism.

The PCRs were carried out by a Veriti Thermal Cycler (Applied Biosystems, USA) in which the DNA templates were denatured at 95 °C for 5 min. The amplification step consisted of 40 cycles of 45 s at 95 °C, 60 s at 59 °C (for *OMENTIN* V109D SNP) and 45 s at 56 °C (for *FTO* rs9939609 SNP), in addition to 72 °C for 45 s with a final extension of 5 min at 72 °C.

Afterward, the PCR products of *Omentin* V109D and *FTO* rs9939609 polymorphic regions were subjected to restriction enzyme analysis by digestion at 37 °C for 8 h with AccI (XmiI) and ScaI restriction endonucleases, respectively (ThermoFisher Scientific, USA). Within the exon-4 of *Omentin* gene, the Val encoded by polymorphic codon GTC can recognize AccI, whereas the GAC codon encoding Asp eliminates the AccI recognition site. In addition, ScaI enzyme recognized the single nucleotide polymorphism T to A in the first intron of *FTO* gene.

In the RFLP tests, each restriction endonuclease mixture with a total volume of 20 μl contained 5 μl amplified fragments, 12 μl DNase free distilled water, 2 μl appropriate buffer, and 1 μl of each restriction endonuclease. One unit of restriction enzyme is the amount of enzyme required to digest 1 μg of lambda DNA in 1 h at 37 °C for AccI and ScaI. The restriction products were separated by electrophoresis on a 3% agarose gel (Sinaclon, Iran) in TBE buffer, and then stained with DNA safe stain.

### Statistical analyses

Statistical analysis was performed in SPSS software (version 18). The demographic and metabolic data were compared between the study groups using the t-test. All the data were expressed as mean and standard deviation. Comparison of the allele frequencies and genotype distribution between the case and control groups was performed using the Pearson’s Chi-squared test. In addition, the correlation between genotype alleles with chemicals and insulin levels was examined by Pearson correlation test. The Hardy-Weinberg equilibrium was assessed using http://scienceprimer.com/hardy-weinberg-equilibrium-calculator.

The Chi-square goodness of fit test was carried out to evaluate allele and genotype frequencies in the case and control groups. Moreover, the allele frequency of SNPs in the study groups was evaluated using odds ratio (OR) without adjusting for confounding factors, such as BMI. *P*-value less than 0.05 was considered statistically significant. The power calculation of this study was estimated at about 85%.

## Results

### Demographic and laboratory analysis

The demographic characteristics and biochemical profile for both healthy controls (*n* = 85) and T2D patients (*n* = 83) are shown in Table 1. The individuals in the case group had a familial history of diabetes and significantly higher weight, FBS, HOMA-IR, and TG, compared to the controls. In addition, the case group was hypertensive (Table 1). Considering the optimal HOMA-IR threshold of 1.85–1.95 reported by Esteghamati et al. [27] for the Iranian population, in the present study, 50.6 and 45.8% of the diabetic patients had moderate and severe IR with the HOMA-IRs of 2–4.5 and > 4.5, respectively. In addition, 36.5% of the healthy controls had a HOMA index of 2–4.5, and 6% of them had HOMA-IR of > 4.5 known as severe IR (OR: 34.78, 95% CI: 22.28–46.75, *P* = 0.0001; Table 2).

**Table 1:** Anthropometrical characteristics and laboratory tests from participating individuals

Parameters ^a^	Case (*n* = 83)	Control (*n* = 85)	*P* value
Age (years)	50.95 ± 11.84	50.51 ± 11.7	0.806
Gender (female %)	63.8%	64.7%	0.909
Familial history of diabetes (%)	77.1%	32.9%	0.0001
Hypertension (%)	51.8%	17.6%	0.0001
Smoking (%)	26.5%	14.1%	0.055
BMI	28.26 ± 2.81	24.55 ± 1.39	0.001
FBG (mmol/L) ^b^	9.45 ± 2.56	4.99 ± 0.54	0.0001
Insulin (μIU/ml) ^b^	13.45 ± 5.97	10.05 ± 4.9	0.094
HOMA-IR^b^	5.39 ± 1.37	2.3 ± 1.89	0.0001
TG (mg/dl) ^c^	221.6 ± 47.7	134.2 ± 28.9	0.0001
Total cholesterol (mg/dl) ^c^	184.4 ± 38.2	158.5 ± 32.0	0.125
LDL (mg/dl) ^c^	97.4 ± 31.2	86.4 ± 24.9	0.083
HDL (mg/dl) ^c^	39.4 ± 10.8	40.4 ± 8.9	0.545
TC/HDL ratio ^c^	4.85 ± 1.4	4.03 ± 0.92	0.096
LDL/HDL ratio ^c^	2.55 ± 0.82	2.20 ± 0.71	0.085
*FTO* rs9939609 polymorphism (% polymorphic allele)	39.2%	20%	0.0001
*Omentin* rs2274907 polymorphism (% polymorphic allele)	27.1%	15.8%	0.011

^a^ Data are expressed as arithmetic mean ± SD

^b^ Diabetes screening tests reference values: Fasting Blood Glucose (FBG) Normal: 3.9–5.5, Prediabetic: 5.6–6.9, Diabetes ≥7; Insulin: 2–25; Homeostatic Model Assessment of Insulin Resistance (HOMA-IR): Normal: < 2.0, Moderate IR: 2.0–4.5, Severe IR: > 4.5;

^c^ Lipid panel reference values: Triglyceride (TG): Normal: < 150, Borderline: 150–199, High: 200–499, Very high: > 500; Total cholesterol (TC): Normal: < 200, Borderline: 200–239, High: > 240; Low density lipoprotein (LDL): Optimal: < 100, Near optimal: 100–129, Borderline: 130–159, High: 160–189, Very high: > 190; High density lipoprotein (HDL): Low: < 40, Normal: 40–60, High: > 60; Total cholesterol/High density lipoprotein ratio (TC/HDL): Low risk < 5, Average risk < 5–7, High risk > 7, Very high risk > 11; Low density lipoprotein/ High density lipoprotein ratio (LDL/HDL): Low risk: < 3.4, Average risk: 3.4–5, High risk > 5

**Table 2:** Frequencies of HOMA-IR severity in study Groups

HOMA-IR (number - %)				
Group	Normal (< 2)	Moderate (2–4.5)	High (> 4.5)	*P* value
Case (Diabetic)	3 (3.6%)	42 (50.6%)	38 (45.8%)	0.0001
Control (Normal)	48 (56.5%)	31 (36.5%)	6 (7%)	

### Distribution of Omentin Val109Asp and FTO rs9939609 polymorphisms

**Fig. 1:**
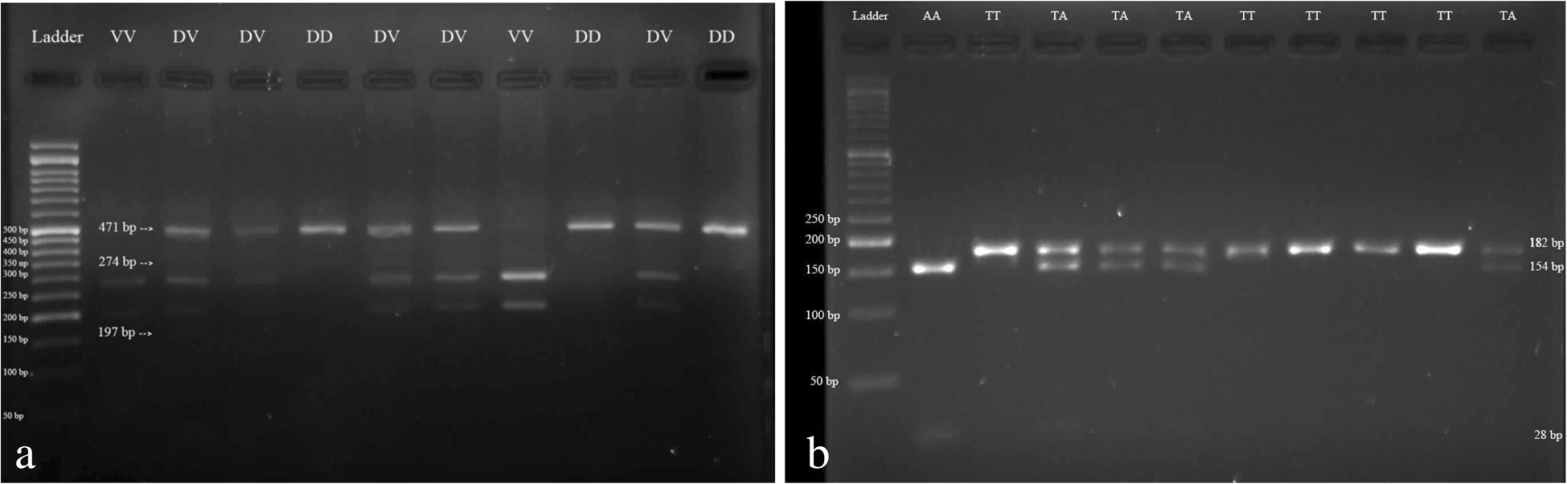
The electrophoretic patterns for determination of *omentin* Val 109 Asp and *FTO* rs9939609 polymorphisms. **a** *Omentin* V109D genotypes include DD (normal), DV (polymorphism in one allele), VV (polymorphism in two alleles). **b** *FTO* rs9939609 genotypes include TT (normal), TA (polymorphism in one allele), AA (polymorphism in two alleles)

Regarding the presence of two alleles in *Omentin* gene, in the determination of *Omentin* V109D polymorphism, the undigested fragment (471 bp) was detected as a healthy homozygote for D allele (genotype DD). In addition, the digested fragments (274 and 197 bp) were identified as homozygotes for V allele (genotype VV). Moreover, both digested and undigested fragments (471, 274, and 191 bp) were detected as heterozygotes (genotype DV; Fig. 1a).

Genotype distribution of *Omentin* V109D polymorphism was in the Hardy-Weinberg equilibrium. Out of 85 controls, 70.6, 27.1, and 2.3% of the individuals were found to have DD, DV, and VV genotypes, respectively. On the other hand, among the 83 cases, DD, DV, and VV genotypes were observed in 48.2, 49.4, and 2.4% of the participants, respectively.

**Fig. 2:**
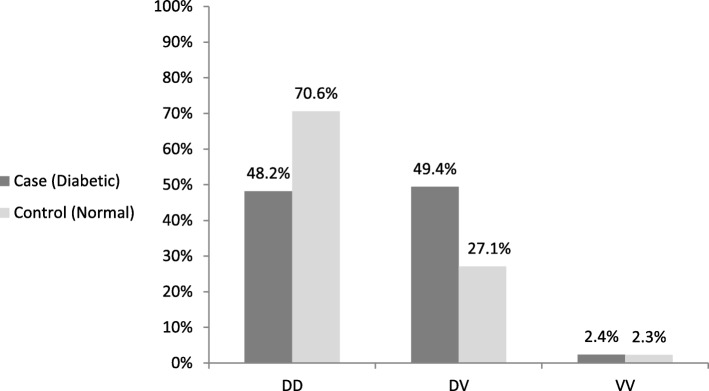
Genotype distribution of *omentin* Val 109 Asp gene polymorphism in study groups. Genotypes include DD (normal), DV (polymorphism in one allele), VV (polymorphism in two alleles)

Based on the results, V allele had the frequencies of 0.158 and 0.271 in the healthy controls and diabetic cases, respectively. There was a significant difference between the case and control groups in terms of relative genotype (*P* = 0.011) and allele frequency (*P* = 0.011; Table 3, Fig. 2). In addition, the presence of the DV and VV genotypes of *Omentin* V109D SNP was associated with a higher risk of T2D (OR = 1.98, 95% CI = 1.35–2.64, *P* = 0.001; Table 3).

**Table 3:** Genotype frequencies of *omentin* V109D and *FTO* rs9939609 polymorphisms in study groups

*omentin* Val 109 Asp polymorphism genotypes (number - %)							*FTO* rs9939609 polymorphism genotypes (number - %)					
Group	DD	DV	VV	D allele (%)	V allele (%)	*P* value	TT	TA	AA	T allele (%)	A allele (%)	*P* value
Case (Diabetic)	40 (48.2%)	41 (49.4%)	2 (2.4%)	72.9%	27.1%	0.011	29 (34.9%)	43 (51.8%)	11 (13.3%)	60.8%	39.2%	0.0001
Control (Normal)	60 (70.6%)	23 (27.1%)	2 (2.3%)	84.2%	15.8%		52 (61.2%)	32 (37.6%)	1 (1.2%)	80%	20%

In terms of *FTO* rs9939609 polymorphism, the undigested fragment (182 bp) was found to be a healthy homozygote (genotype TT) and the digested fragments (154 and 28 bp) were detected as polymorphic homozygotes (genotype AA). Moreover, both digested and undigested fragments (182, 154, and 28 bp) were identified as heterozygotes (genotypes TA) (Fig. 1b).

**Fig. 3:**
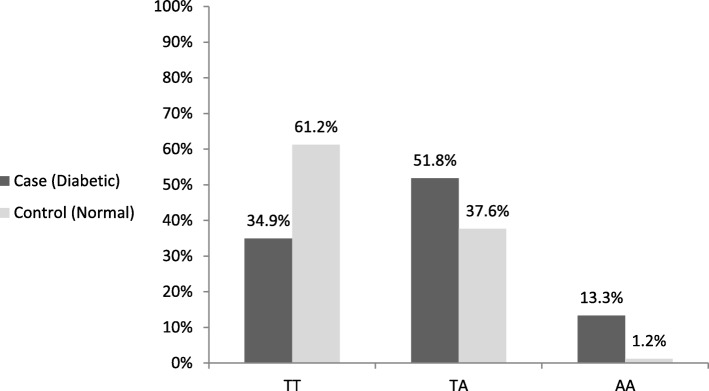
Genotype distribution of *FTO* rs9939609 gene polymorphism in study groups. Genotypes include TT (normal), TA (polymorphism in one allele), AA (polymorphism in two alleles)

The *FTO* rs9939609 genotype frequency, as well as allele frequency is shown in Table 3. The genotype frequency of heterozygosity for TA alleles in the case and control groups was 51.8 and 37.6%, respectively. Moreover, the frequencies of two-allele polymorphism (AA) in the case and control groups were obtained as 13.3 and 1.2%, respectively. Regarding the frequency of A allele, it was estimated at 0.392 and 0.2 in the diabetic cases and healthy controls, respectively (OR = 2.57, 95% CI = 1.87–3.04, *P* = 0.0001; Table 3, Fig. 3).

Concerning the higher prevalence of *Omentin* and *FTO* gene polymorphisms in the subjects with higher fat mass, the investigation of the genotype distribution of these polymorphic regions in the study population would be helpful. Table 4 presents the combinational *Omentin-FTO* genotypes in the study population. In this regard, the included genotypes were DD/TT (DT), DV/TT (DVT), VV/TT (VT), DD/TA (DTA), DV/TA (DVTA), VV/TA (VTA), DD/AA (DA), DV/AA (DVA), and VV/AA (VA). The results showed a significant difference between the two study groups in this respect so that the polymorphic genotypes were more frequent in the diabetic individuals than in the healthy controls (*P* < 0.0001).

**Table 4:** Combinational *omentin-FTO* genotypes in study groups

Groups Combinational genotypes	Case (Diabtic)	Control (Healthy)
DT	18.1%	46.1%
DVT	15.6%	14.2%
VT	1.2%	1.1%
DTA	22.9%	24.7%
DVTA	27.7%	11.7%
VTA	1.2%	1.1%
DA	7.2%	0%
DVA	6.1%	1.1%
VA	0%	0%

### Relationship of Omentin V109D and FTO rs9939609 polymorphisms with IR and other biochemical parameters

Table 5 demonstrates the distribution of genotype/allele frequencies for *Omentin* and *FTO* polymorphisms in the participants according to HOMA-IR index for determining their genetic polymorphism relationship with IR. In terms of *Omentin* V109D SNP, 17.3 and 4.2% of the subjects with increased and normal HOMA index had V polymorphic allele, respectively (OR = 1.78, 95% CI = 1.57–1.96, *P* = 0.03).

**Table 5:** Association between *Omentin* V109D and *FTO* rs9939609 polymorphisms with HOMA-IR in study population

*omentin* Val 109 Asp polymorphism genotypes (number - %)							*FTO* rs9939609 polymorphism genotypes (number - %)					
HOMA-IR	DD	DV	VV	D allele	V allele	*P* value	TT	TA	AA	T allele	A allele	*P* value
HOMA-IR Normal (< 2.0)	38 (22.6%)	12 (7.2%)	1 (0.6%)	26.2%	4.2%	0.030	30 (17.8%)	20 (11.9%)	1 (0.6%)	23.75%	6.55%	0.046
HOMA-IR High (> 2.0)	62 (36.9%)	52 (30.9%)	3 (1.8%)	52.35%	17.25%		51 (30.3%)	55 (32.8%)	11 (6.6%)	46.7%	23%	

Regarding *FTO* rs9939609 SNP, 23% of the participants with a high HOMA index had A polymorphic allele, while 6.6% of the individuals with a normal HOMA index had A polymorphic allele (OR = 2.05, 95% CI = 1.88–2.21, *P* = 0.046). The correlation tests indicated that *Omentin* D109V polymorphism was correlated with higher HOMA-IR (*P* = 0.011), while *FTO* polymorphism was not significantly correlated with IR according to HOMA-IR (*P* = 0.062). Furthermore, there was no correlation between *Omentin* V109D and *FTO* polymorphisms (*P* = 0.084).

The results showed that 21.8% of the participants with overweight/obesity had *FTO* A allele, while 7.8% of the individuals with normal BMI had *FTO* A allele. There was no significant genotype-related difference between the study groups with high BMI concerning the *FTO* polymorphism (*P* = 0.383). In addition, 17 and 4.5% of the subjects with overweight/obesity and normal BMI had *Omentin* V allele, *Omentin* respectively (*P* = 0.048). The results of the Pearson correlation test showed that *FTO* polymorphism was not correlated with high BMI (*P* = 0.054), while *Omentin* polymorphism was correlated with higher BMI (*P* = 0.002).

The frequency distributions of *FTO* rs9939609 polymorphic A allele were estimated at 19.4 and 10.1% in the individuals with a familial history of diabetes and those without the history, respectively (*P* = 0.024). Moreover, the *Omentin* V allele was significantly correlated with the familial history of diabetes (14.3% for cases vs. 7.2% for controls; *P* = 0.046). On the other hand, the frequency distributions of *FTO* A allele were 12.5 and 16.9% in the hypertensive and normotensive participants, respectively (*P* = 0.099).

Furthermore, the *Omentin* V allele did not have a significant correlation with hypertension (9.2% for cases vs. 12.2% for controls; *P* = 0.179). Concerning the relationship between the *Omentin* and *FTO* genotypes, as well as between allele frequencies and lipid profile (e.g., TG, TC, HDL-C, and LDL-C), no significant difference was found between the polymorphic alleles and the lipid profile.

## Discussion

Central obesity and accumulation of visceral adipose tissue are known as risk factors for IR development and subsequent T2D [4, 5]. Diabetes mellitus and IR are affected by genetic factors, as well as acquired overweight or obesity. As a result, it is important to find the gene polymorphisms related to adipose tissues, especially in people with a familial history of diabetes.

The relationship of *Omentin* Val 109 Asp and *FTO* rs9939609 genetic variations with newly diagnosed diabetes is not well determined yet. Consequently, we investigated these polymorphisms in some patients with T2D from the Northeast of Iran for the first time through clinical examinations and laboratory tests. The results of the present study revealed significant relationship between both *FTO* rs9939609 and *Omentin* Val 109 Asp polymorphisms with T2D in the studied population (*P* = 0.0001 and *P* = 0.011, respectively).

Moreover, our findings through HOMA-IR indicated that both gene polymorphisms of *FTO* rs9939609 (T/A) and *Omentin* rs2274907 (Val 109 Asp) were significantly related to IR (*P* = 0.046, *P* = 0.03, respectively). In terms of BMI, the *Omentin* V109D polymorphism was observed to have a significant relationship with overweight/obesity. In addition, both *FTO* rs9939609 and *Omentin* Val 109 Asp polymorphisms had a significant positive correlation with a familial history of diabetes (*P* = 0.024 and *P* = 0.046, respectively).

Several studies have investigated the relationship of Val 109 Asp polymorphism in *Omentin* gene and/or Omentin-1 levels with different diseases, such as diabetes [11], rheumatoid arthritis [28], psoriasis [29], osteoporosis [30], and coronary artery disease [31]. Some studies have shown that circulating Omentin-1 may enhance glucose uptake in human adipocytes by insulin via Akt signaling [8–10].

de Souza Batista et al. suggested that the plasma level of Omentin-1 was inversely correlated with BMI, waist circumference, leptin levels, and HOMA-IR. However, it was positively correlated with adiponectin and HDL levels. Moreover, they reported the downregulation of both *Omentin 1* and *Omentin 2* genes in obese people [5]. Although we did not measure the plasma Omentin-1 in the present study, it was observed that the A326T (rs2274907) genetic variation in the *Omentin 1* gene could have a relationship with higher BMI, as well as IR.

Some studies have revealed an inverse relationship between plasma Omentin-1 levels and the obesity and IR [5]. Moreover, some researches showed that *Omentin* Val 109 Asp gene polymorphism is correlated with obesity and diabetes mellitus [11, 32]. It seems that Asp substitution by Val in position 109 of Omentin-1 might diminish the activity and/or lifetime of circulating Omentin-1. Therefore, concerning the positive effect of Omentin on glucose uptake by insulin receptors [8], the Val 109 Asp gene polymorphism may be inversely related to plasma Omentin and insulin levels/functions.

Various studies indicated that *Omentin* Val109Asp gene polymorphism is associated with T2D [11, 33]. Nonetheless, this genetic variation and its correlation with IR have not been investigated in patients newly diagnosed with T2D. According to the results of the present study, the polymorphic V allele of *Omentin* gene was correlated to two critical factors for T2D development, namely IR and BMI. Consequently, this polymorphism was found to be associated with T2D occurrence.

Schäffler et al. conducted a study on Caucasian patients with T2D or chronic inflammatory bowel disease (IBD). They stated that V allele was present in 26% of the healthy controls, while 30 and 31% of the patients with T2D and IBD had V allele, respectively. On the other hand, they did not show any significant difference in genotype distribution between the study groups. Furthermore, in the mentioned study, no significant relationship was found between the genotype subgroups and the anthropometric and laboratory parameters in diabetic patients [11].

Mrozikiewicz-Rakowska et al. reported that the rs2274907 (V 109 D) variant of *Omentin* gene is associated with the elevated prevalence of diabetic foot in patients with diabetes mellitus. However, they did not observe a significant difference in the distribution of alleles between the diabetic individuals with and without diabetic foot [33]. Our results also showed a significant difference between the study groups in terms of *Omentin* V allele.

In this study, the genotype distributions of Asp/Val were 50 and 34.3% in the diabetic patients and healthy controls, respectively. In addition, the genotype distributions of Val/Val were obtained as 3.8 and 1.4% in the diabetic patients and healthy controls, respectively. Moreover, statistical analysis confirmed that the probability risk for T2D in individuals who had V allele was 1.98 times higher than that for people with normal allele.

Bahadori et al. concluded that Iranian women with Val/Val genotype had a greater risk of obesity complications in comparison to those with Asp/Asp [32]. Isakova et al. investigating Kyrgyz population reported that the Val/Val genotype was significantly more prevalent in the patients with abdominal obesity than in the healthy controls (OR = 3.12). However, the allelic variants of *Omentin* Val 109 Asp polymorphism did not correlate with abdominal obesity [34].

In line with the latter results, our data indicated that Val/Val homozygous genotype was more frequent in overweight or obese people. In addition, there was a significant relationship between the allelic genotypes of *Omentin* Val 109 Asp polymorphism and BMI. Kohan et al. investigated the influence of *Omentin* V109D polymorphism on genetic susceptibility to nonalcoholic fatty liver disease in Iranian population. They demonstrated that the frequency of *Omentin* V 109 D gene polymorphism in these patients was significantly different from that of the healthy controls (OR = 2.3) [35].

It is hypothesized that *Omentin* Val 109 Asp gene polymorphism may have a role in lipid accumulation in the liver. Furthermore, Omentin levels and the V 109 D polymorphism are in association Omentin with BMI and fatty liver disease. Therefore, further studies concerning the unknown mechanism of Omentin effect on lipogenesis and lipid accumulation may render helpful results.

Several studies indicated that the fat mass and obesity-associated (*FTO*) gene, especially the *FTO* rs9939609 variant, has a relationship with body weight and fat mass [12, 15, 36–39]. However, some studies in Asia did not find any relationship between *FTO* rs9939609 A polymorphic allele and overweight [16, 40]. The *FTO* rs9939609 polymorphism is less frequent in East Asians (12.6%) [38], compared to that in Europeans (45%) and West Africans (52%) [12, 14].

Although some studies showed a significant relationship between *FTO* rs9939609 variant and T2D [41], others did not find any relationship [38]. In addition, the *FTO* variant is correlated with a higher occurrence of T2D. However, this effect can be entirely explained by the BMI differences between the diabetic and healthy controls [12, 42, 43].

Chey et al. performed a study on Malaysian multi-ethnic population, including Malays, Chinese, and Indians. They concluded that the genotype distribution and allele frequencies of *FTO* rs9939609 gene variant were significantly different among the ethnicities. On the other hand, they did not observe any significant relationship between *FTO* polymorphism and obesity [16].

In the present study, the relationship between *FTO* rs9939609 gene variant and higher BMI was not significant, which is consistent with the findings of the mentioned study. However, our results showed that this polymorphism was correlated with IR. Moreover, the results of the current study demonstrated that the participants with A allele had a ~ 2.6-fold higher susceptibility to T2D than the control subjects.

Freathy et al. indicated that *FTO* rs9939609 A allele is correlated with higher fasting insulin, FBS, TG, and lower HDL-C. They concluded that *FTO* polymorphism has a relationship with metabolic syndrome (OR = 1.17). It is noteworthy that they found no evidence of the mentioned relationships when adjusted for BMI. Moreover, no significant relationship was observed between *FTO* polymorphism and serum alanine aminotransferase, γ-glutamyl-transpeptidase, LDL-C, HbA1c, and systolic and diastolic blood pressure [7]. In accordance with the mentioned study, our findings demonstrated that *FTO* A allele is correlated with higher FBS in newly diagnosed T2D patients (*P* = 0.0001). However, there was no significant relationship between this polymorphic allele and lipid profile.

Chang et al. confirmed the role of *FTO* genetic variants in obesity and T2D in the Chinese population by a genome-wide study on 19 SNPs. They found that the rs9939609 A allele was strongly correlated with obesity and higher BMI. However, they did not observe significant relationships between 19 SNPs with T2D or other obesity-related traits [14]. In contrast to the results obtained by Chang et al., our findings demonstrated no significant correlation between rs9939609 A allele and higher BMI. In addition, our data showed that *FTO* A allele is associated with IR and T2D. These controversial data confirm the effect of ethnicity in distinct populations on *FTO* rs9939609 polymorphism.

The results of the present study demonstrated that *FTO* rs9939609 polymorphism has a relationship with the familial history of diabetes (*P* = 0.024), IR (*P* = 0.046), and T2D (*P* = 0.0001) in Northeast Iranian population. However, no relationship was detected between *FTO* A allele and higher BMI.

Our data showed that each additional *FTO* A allele could alter the risk of diabetes with an OR of 2.6 and 95% CI of 1.87–3.04 when the case and control subjects are not matched in terms of BMI. The *FTO* A allele had a frequency of 39.2 and 20% *FTO* in diabetic patients and healthy people, respectively. In addition, 23% of the individuals with a higher HOMA index considered as moderate or severe IR had A allele. This might be indicative of the role of *FTO* rs9939609 A allele in IR. Therefore, further studies are required to examine whether *FTO* polymorphism alters insulin secretion or targets tissue response to insulin.

One of the limitations of the present study is its relatively small sample size. As a result, the frequency of genetic polymorphisms should be confirmed by further investigations in a larger population. It should be mentioned that in the current study, sample size was selected according to the frequency of T2D among the population residing in Bojnurd. In addition, it was difficult to find new diabetic patients to exclude the probable effects of anti-diabetic drugs on study gene polymorphisms and other clinical or biochemical variables, compared to other studies focused on gene polymorphisms in patients with overt diabetes.

## Conclusion

The findings of this study demonstrated that 96.4% of the newly diagnosed Iranian patients with T2D had IR as estimated by HOMA-IR index. Moreover, the statistical analysis showed that the probability risk of disease in individuals with IR was 34.8 times higher than that in the subjects with normal HOMA index. In the current study, both *Omentin* V109D and *FTO* rs9939609 polymorphisms had a relationship with IR and familial history of diabetes in the newly diagnosed T2D patients.

In addition, the *Omentin* V109D polymorphism (not *FTO* rs9939609) was related to higher BMI. Moreover, combinational genotyping confirmed that DV/TA (one polymorphic allele in each studied gene), DD/AA (two polymorphic alleles of *FTO* versus normal *Omentin* genotype), and DV/AA (one polymorphic allele of *Omentin* and two polymorphic alleles of *FTO*) genotypes were more frequent in the diabetic patients than in the healthy controls.

These results suggested that *Omentin* V 109 D and *FTO* rs9939609 genetic variations may change insulin metabolism, especially via the target tissue receptors leading to T2D development through IR. Therefore, the evaluation of these polymorphic regions may be helpful for predicting T2D and the related morbidities in normal subjects with a familial history of diabetes and high HOMA index. As a result, the implementation of genome-wide association studies on more gene polymorphisms related to IR and familial histories of diabetes is an efficient strategy by determining the newly diagnosed diabetic patients and prevent diabetic morbidities.

## Data Availability

The authors confirm that all of the data and material of the manuscript are available for more consideration by the journal.

## Abbreviations

BMI: Body mass index
FBS: Fasting blood sugar
FTO: Fat mass and obesity associated
HDL-C: High density lipoprotein-cholesterol
HOMA-IR: Homeostasis model assessment - estimated insulin resistance
IR: Insulin resistance
LDL-C: Low density lipoprotein-cholesterol
PCR: Polymerase chain reaction
RFLP: Fragment length polymorphism analysis
T2D: Type 2 diabetes
TC: Total cholesterol
TG: Triglycerides
